# Lower Humoral and Cellular Immunity Following Asymptomatic SARS-CoV-2 Infection Compared to Symptomatic Infection in Education (The ACE Cohort)

**DOI:** 10.1007/s10875-024-01739-0

**Published:** 2024-06-10

**Authors:** Georgina Hopkins, Nancy Gomez, Davis Tucis, Laura Bartlett, Graham Steers, Ellie Burns, Michaela Brown, Tyler Harvey-Cowlishaw, Rute Santos, Sarah N Lauder, Martin Scurr, Lorenzo Capitani, Stephanie Burnell, Tara Rees, Kathryn Smart, Michelle Somerville, Awen Gallimore, Marianne Perera, Martin Potts, Marina Metaxaki, Benjamin Krishna, Hannah Jackson, Paddy Tighe, David Onion, Andrew Godkin, Mark Wills, Lucy Fairclough

**Affiliations:** 1 School of Life Sciences, University of Nottingham, Nottingham, UK; 2 School of Medicine, Cardiff University, Cardiff, UK; 3 Department of Medicine, University of Cambridge, Cambridge, UK; 4 ImmunoServ Ltd, Cardiff, UK

**Keywords:** Asymptomatic, Symptomatic, SARS-CoV-2, Vaccination, Antibody, T cells

## Abstract

**Purpose:**

Asymptomatic SARS-CoV-2 infections were widely reported during the COVID-19 pandemic, acting as a hidden source of infection. Many existing studies investigating asymptomatic immunity failed to recruit true asymptomatic individuals. Thus, we conducted a longitudinal cohort study to evaluate humoral- and cell-mediated responses to infection and vaccination in well-defined asymptomatic young adults (the Asymptomatic COVID-19 in Education [ACE] cohort).

**Methods:**

Asymptomatic testing services located at three UK universities identified asymptomatic young adults who were subsequently recruited with age- and sex-matched symptomatic and uninfected controls. Blood and saliva samples were collected after SARS-CoV-2 Wuhan infection, and again after vaccination. 51 participant’s anti-spike antibody titres, neutralizing antibodies, and spike-specific T-cell responses were measured, against both Wuhan and Omicron B.1.1.529.1.

**Results:**

Asymptomatic participants exhibited reduced Wuhan-specific neutralization antibodies pre- and post-vaccination, as well as fewer Omicron-specific neutralization antibodies post-vaccination, compared to symptomatic participants. Lower Wuhan and Omicron-specific IgG titres in asymptomatic individuals were also observed pre- and post-vaccination, compared to symptomatic participants. There were no differences in salivary IgA levels. Conventional flow cytometry analysis and multi-dimensional clustering analysis indicated unvaccinated asymptomatic participants had significantly fewer Wuhan-specific IL-2 secreting CD4^+^ CD45RA^+^ T cells and activated CD8^+^ T cells than symptomatic participants, though these differences dissipated after vaccination.

**Conclusions:**

Asymptomatic infection results in decreased antibody and T cell responses to further exposure to SARS-CoV-2 variants, compared to symptomatic infection. Post-vaccination, antibody responses are still inferior, but T cell immunity increases to match symptomatic subjects, emphasising the importance of vaccination to help protect asymptomatic individuals against future variants.

**Supplementary Information:**

The online version contains supplementary material available at 10.1007/s10875-024-01739-0.

## Introduction

In 2020, the World Health Organisation declared the novel coronavirus, severe acute respiratory syndrome coronavirus 2 (SARS-CoV-2), outbreak a global pandemic, subsequently accounting for an estimated 18.2 million deaths worldwide between January 2020 and December 2021 [1]. The high rate of transmission and infection was the catalyst of the pandemic, resulting in the implementation of infection control measures, such as the isolation of individuals experiencing symptoms [2]. The proportion of those with no symptoms during SARS-CoV-2 infection, termed asymptomatic, was estimated to be 20-44% of COVID-19 cases [3–7], with increased cases in children to young adults, but decreased asymptomatic cases at older ages [7]. With a high frequency of asymptomatic cases, in addition to symptomatic individuals transmitting SARS-CoV-2 before the onset of symptoms, SARS-CoV-2 was transmitted silently, exacerbating the pandemic.

The assessment of the immune response to SARS-CoV-2 infection has primarily focused on spike-specific antibody responses or neutralising titres [8, 9]; SARS-CoV-2-infected individuals display neutralising antibodies for months or even years, facilitating these measurements [10]. The challenges of cellular assays, and logistics of obtaining suitable samples, has led to fewer studies focused on measuring specific T cell responses to SARS-CoV-2. T cells regulate antibody (humoral) responses, but also mediate a faster and more potent response upon further exposure to viral antigens, underpinning a long-lasting immunity and vaccine efficacy [11]. Indeed, there is evidence that cognate T cell responses are a better indicator of immunity than antibody levels [12]. T cell memory after SARS-CoV-2 infection is established with robust CD4^+^ and CD8^+^ T cell responses to a combination of spike, membrane, and nucleocapsid viral proteins [13], or spike-specific responses after vaccination.

The question of whether humoral and/or cellular immunity following asymptomatic SARS-CoV-2 infection is sufficient to protect from future strains compared to symptomatic infection is uncertain. Existing evidence suggests asymptomatic infection results in a faster decline of SARS-CoV-2-specific T cells compared to symptomatic infection [14], indicating a relationship between increased memory T cells and symptomatic disease. However, there is also evidence for a more robust SARS-CoV-2-specific early T cell-mediated response in asymptomatic than symptomatic patients, but a weaker neutralising antibody profile [15, 16].

Developing an understanding of asymptomatic and symptomatic immune responses to SARS-CoV-2 is imperative in minimising the future impact of another public health threat, such as through the development of T cell-targeted vaccines for any future strains of SARS-CoV-2 that may develop. In addition, understanding cross-reactivity to new variants is key for planning for potential vaccine strategies. This study took advantage of an on-going asymptomatic screening service developed at three universities in the UK, allowing measurements of T cell and serological responses to SARS-CoV-2 in individuals with true asymptomatic infection. These samples enabled us to build upon existing research and understand for the first time the immune responses in this asymptomatic, largely young, healthy demographic, with matched symptomatic subjects, following SARS-CoV-2 natural infection and subsequent vaccination. Utilising this genuine asymptomatic cohort, we identify key decreases in both humoral and cellular immune responses to SARS-CoV-2 in asymptomatic infection compared to symptomatic infection.

## Methods

### Study Design and Cohort

A multi-centre longitudinal cohort study was conducted in individuals identified as part of three University Asymptomatic Testing Services (The University of Nottingham, The University of Cambridge and Cardiff University). Eligible participants were aged 18 years or older. Participants were excluded if they were under 18 years old, unable to provide blood samples, had low English proficiency, unable to travel for the study visits, or unable to provide informed consent. Young adults who experienced no symptoms but were identified as positive for SARS-CoV-2 infection (asymptomatic) by one of the asymptomatic testing services, utilising antibody and PCR tests, were recruited into the study. Age and sex-matched participants, who were either negative for SARS-CoV-2 infection (had no symptoms and a negative test result for SARS-CoV-2), or who had symptomatic infection (experienced symptoms of SARS-CoV-2 infection and received a positive test result) were also recruited into the study.

### Data and Sample Collection

A baseline questionnaire including information on demographics, clinical factors (previous COVID-19 infection and tests), and vaccination details was completed. Blood samples to isolate peripheral blood mononuclear cells (PBMCs) and plasma, as well as saliva samples, were then collected from all participants in Spring 2021, pre-vaccination, and a minimum of 1-week post-infection for asymptomatic and symptomatic participants. The participants then returned in Summer 2021, post-vaccination for a second blood and saliva collection. Whole blood samples were collected into heparin vacutainers (Greiner Bio-One) and within 4 hours they were diluted with an equal volume of PBS + 1% foetal bovine serum (FBS) and separated by density-gradient centrifugation, using Histopaque solution (Sigma) with SepMate tubes (Stem cell Technologies). PBMCs were washed twice and then immediately cryopreserved at -80°C in 10% DMSO and 90% FBS at a cooling rate of 1°C/min and subsequently transferred to liquid nitrogen storage (-196°C) until experimental use. Saliva samples were collected by spitting into a tube which contained a 2% final concentration Triton X-100 and stored at -80°C until use in IgA antibody ELISAs.

### Antibody Titre Assay

Heparinized whole blood was centrifuged at 300 xg for 8 minutes and the upper plasma containing layer removed and further centrifuged at 800 xg for 5 minutes. Plasma was tested using two separate ELISAs for detection of Wuhan and Omicron-specific spike antibodies. Full methods can be found in supplementary methods.

### Salivary IgA Assay

Before performing ELISAs, saliva samples were centrifuged at 1200 rpm for 1 minute, and a visual quality control step was performed, removing samples with excess debris present. IgA ELISAs were then performed (details found in supplementary methods), with samples diluted 1:15 in 3% whey blocking solution, replacing gamma-chain specific anti-human IgG HRP conjugate with an alpha-chain specific anti-human IgA HRP conjugate (Sigma, A0295), at a 1:10,000 dilution.

### Generation of Pseudotyped Lentiviral Particles

Assays were performed as previously described [17]. In brief, Wuhan and Omicron pseudotyped viruses were generated using triple plasmid transfection of HEK293T cells with spike-expressing plasmid along with the lentiviral packaging vector p8.91 and luciferase expression vector psCSFLW using Lipofectamine transfection reagent (Thermo-Fisher). Lentiviruses were harvested after 48 h, passed through a 0.45μm filter, stored in aliquots at -80°C and titrated on HeLa cells expressing ACE-2 (HeLa-ACE2).

### Virus Neutralization Assays

As shown previously [17], pseudotyped viruses were neutralized by incubating with serially diluted, heat-inactivated human plasma samples for 1 h at 37°C. Full methods can be found in supplementary methods.

### T Cell Stimulation With Overlapping Peptide Pools

Cryopreserved PBMCs were thawed, washed, and resuspended in RPMI1640 10% FBS, 10U/mL benzonase (1 hr, 37C). Cells were washed and resuspend at 8x10^6^ cells/ml in RPMI + 5% human AB serum in 96 well plates. PBMCS were stimulated with 0.6nM (~1mg/ml) SARS-CoV-2 overlapping peptide pools for Wuhan (Miltenyi) or Omicron (B.1.1.529.1) (Proimmune) spike. As positive controls cells were stimulated with 50 ng/mL Phorbol myristate acetate (PMA) and 0.5 μg/mL Ionomycin (for ICS assay), or 1 μg/mL Phytohemagglutinin (PHA) (for activation-induced marker (AIM) assay). As peptide pools were reconstituted in high-grade pure sterile H_2_O, cells incubated with a matched volume of H_2_0 were used as a negative control. PBMCs were then split into either the AIM assay or ICS assay below.

### Activation-Induced Marker Assay

An AIM assay was used to identify total CD4^+^ and CD8^+^ T cells responding to spike. cells were stimulated with peptides for 24 hours to allow adequate activation and stained with viability dye and surface markers (Table 1, 30 mins 4 °C). Cells were washed and resuspended in 4% paraformaldehyde before analysis on a Sony ID7000 spectral cell analyzer. The gating strategies for the AIM assay can be found in supplementary Figure 1.

**Table 1:** Flow cytometry panel for AIM markers. Extracellular markers utilised for T cell activation assay

Cell Type	Marker	Fluorochrome	Clone	Manufacturer	RRID
Dead cells	-	Zombie Aqua / BV510	-	Biolegend	-
T cells	CD3	PerCP-Cy5.5	SK7	Biolegend	AB_10640736
CD4^+^ T cells	CD4	APC-Fire750	SK3	Biolegend	AB_2572097
CD8^+^ T cells	CD8	Alexa Fluor (AF) 700	RPA-T8	Biolegend	AB_493745
Monocytes	CD14	BV605	63D3	Biolegend	AB_2716231
B cells	CD19	BV605	HIB19	Biolegend	AB_2562015
T cell memory phenotype	CD45-RA	AF488	HI100	Biolegend	AB_528816
	CD27	PE-Cy7	O323	Biolegend	AB_2561919
Activation markers	CD69	PE/Fire 640	FN50	Biolegend	AB_2888777
	CD137	APC	4B4-1	Biolegend	AB_830672
	CD134	PE	Ber-ACT35	Biolegend	AB_10645478

### Intracellular Staining Assay

An intracellular staining (ICS) assay was performed to determine the memory phenotype and cytokine profile of sars-co2 specific T-cells. For the ICS assay, 1x protein transport inhibitor cocktail (Thermofisher) was added after two hours of peptide stimulation and cells were cultured for an additional 4 hours (37°C, 5% CO_2_). A 21-colour flow cytometry panel was used (Table 2). Surface markers were stained for 30 minutes at 4 °C), then washed and fixed with 4% paraformaldehyde (15mins, 4°C), before being washed and permeabilized with permeabilization wash buffer Cells were then stained for intracellular cytokines in the presence of permeabilization wash buffer (30 mins, RT). Cells were then washed and fixed with 4% paraformaldehyde before analysis on a Sony ID7000 spectral cell analyzer.

**Table 2:** Extracellular and Intracellular Flow Cytometry Markers for SARS-CoV-2 Immune Responses. Fluorescent-conjugated antibodies for T cell phenotyping and cytokine profiling were utilised

Cell Type	Target	Fluorochrome	Clone	Manufacturer	RRID
Dead cells	Live/Dead	Zombie Aqua / BV510	-	Biolegend	-
T cells	CD3	PerCP-Cy5.5	SK7	Biolegend	AB_10640736
CD4^+^ T cells	CD4	APC-Fire750	SK3	Biolegend	AB_2572097
CD8^+^ T cells	CD8	Alexa Fluor (AF) 700	RPA-T8	Biolegend	AB_493745
Monocytes	CD14	BV605	63D3	Biolegend	AB_2716231
B Cells	CD19	BV605	HIB19	Biolegend	AB_2562015
T cell memory phenotype	CD27	PE-Cy7	O323	Biolegend	AB_2561919
	CD28	PE/Dazzle 594	CD28.2	Biolegend	AB_2564235
	CCR7	BV421	G0437	Biolegend	AB_11203894
	CD45-RA	AF488	HI100	Biolegend	AB_528816
NK cells and NKT cells	CD56	BV 785	5.1H11	Biolegend	AB_2566059
Senescence/ exhaustion phenotype	CD57	Efluor45D	TB01	Thermofisher	AB_2016680
	CD152 (CTLA-4)	PE-Cy5	BNI3	BD Biosciences	AB_396177
	CD279 (PD-1)	BV750	EH12.2H7	Biolegend	AB_2810505
T cell activation	CD69	PE/Fire 640	FN50	Biolegend	AB_2888777
	CD95	BUV 395	DX2	BD Biosciences	AB_2740044
	CD38	BV 650	HB7	Biolegend	AB_2566233
	HLA-DR	BV 711	L243	Biolegend	AB_2562913
Cytokines	IFN-g	APC	B27	BD Biosciences	AB_398580
	IL-2	PE	JES6-5H4	Biolegend	AB_315302
	TNF-a	PE-eFluor 610	MAB11	Thermofisher	AB_2574667

The gating strategies for the ICS assay can be found in supplementary Figure 2.

### Multi-dimensional Clustering Analysis

To fully elucidate the differences between phenotypes of spike specific T-cells between asymptomatic and symptomatic infection, multi-dimensional clustering analysis was performed. Single, viable, CD3^+^/CD14^-^/CD19^-^ T-cells from spike peptide-stimulated PBMCS were gated in FlowJo (V.10) and 68 thousand T-cells down sampled per donor. T-cells from 19 donors within each group (asymptomatic unvaccinated, asymptomatic vaccinated, symptomatic unvaccinated, and symptomatic vaccinated) were concatenated and cytokine producing cells gated for by unsupervised analysis. For visualization, T-distributed stochastic-neighbour embedding (t-SNE) was performed with opt-SNE learning configuration, 1000 iterations, a perplexity of 30, learning rate of 3500, Exact (vantage point tree) KNN and Barnes-Hut gradient algorithm [18]. FlowSOM clustering [19] was performed using 28 meta-clusters for Wuhan analysis, and 27 meta-clusters for Omicron analysis, and projected on the t-SNE using Cluster Explorer.

### Statistical Analysis

Details on data analysis can be found in the supplementary methods.

**Fig. 1:**
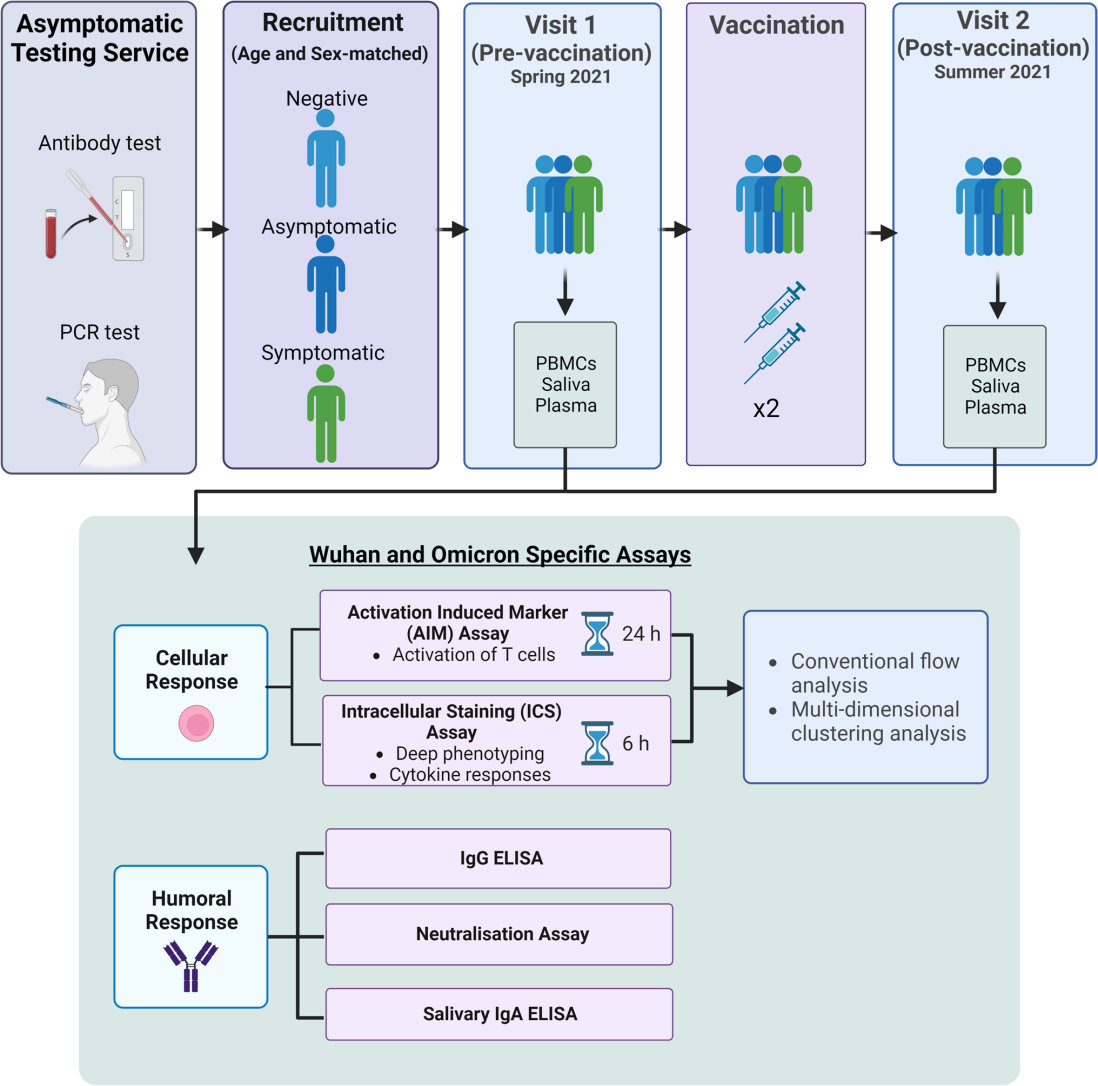
Study design for the recruitment of participants and analysis of humoral and cellular responses to SARS-CoV-2. Asymptomatic young adults were identified by the asymptomatic testing service across 3 universities in the UK, utilising antibody and PCR tests. Asymptomatic participants were then recruited into the study with age and sex-matched participants. Samples were obtained before and after vaccination, then Wuhan and Omicron-specific assays were conducted. Created using BioRender

A summary of the study design and methodology is illustrated in Fig. 1.

## Results

### Donor Characteristics

The demographics of participants is shown in Table 3. Samples were grouped according to infection status (negative, asymptomatic or symptomatic infection) and were taken from the same donors at two time points (one prior to vaccination with an infection date pre-August 2021 (unvaccinated) and one following two vaccinations (vaccinated)). All asymptomatic and symptomatic participants were infected with the Wuhan or Alpha variant. There were no significant differences in age, ethnicity, days since vaccination, vaccine type, or days between visits, between groups (*p* > 0.05) (statistical information can be found in supplementary Table 1).

**Table 3:** Demographics of Participants. The sex, age, ethnicity, days since vaccination, type of vaccine received, and days between visit 1 and visit 2, of 51 participants

	Negative (%)	Asymptomatic (%)	Symptomatic (%)
Sex			
Male	4 (44%)	7 (30%)	10 (53%)
Female	5 (66%)	16 (70%)	9 (57%)
Age			
Median	22	26	21
Range	19-27	18-40	18-55
Ethnicity			
White	9 (100%)	17 (74%)	8 (42%)
Non-white	0 (0%)	4 (17%)	6 (32%)
Unknown	0 (0%)	2 (9%)	5 (26%)
Days since Vaccination			
Median	66	75	99.5
Range	17-146	14-192	27-189
Vaccine Type			
Pfizer	7 (78%)	12 (52%)	12 (63%)
AstraZeneca	1 (11%)	11 (48%)	3 (16%)
Moderna	1 (11%)	0 (0%)	2 (10.5%)
Unknown	-	-	2 (10.5%)
Days between Visits			
Median	206	236	217
Range	106-217	128-282	22-293

### Wuhan and Omicron Specific-Antibody Profiles

**Fig. 2:**
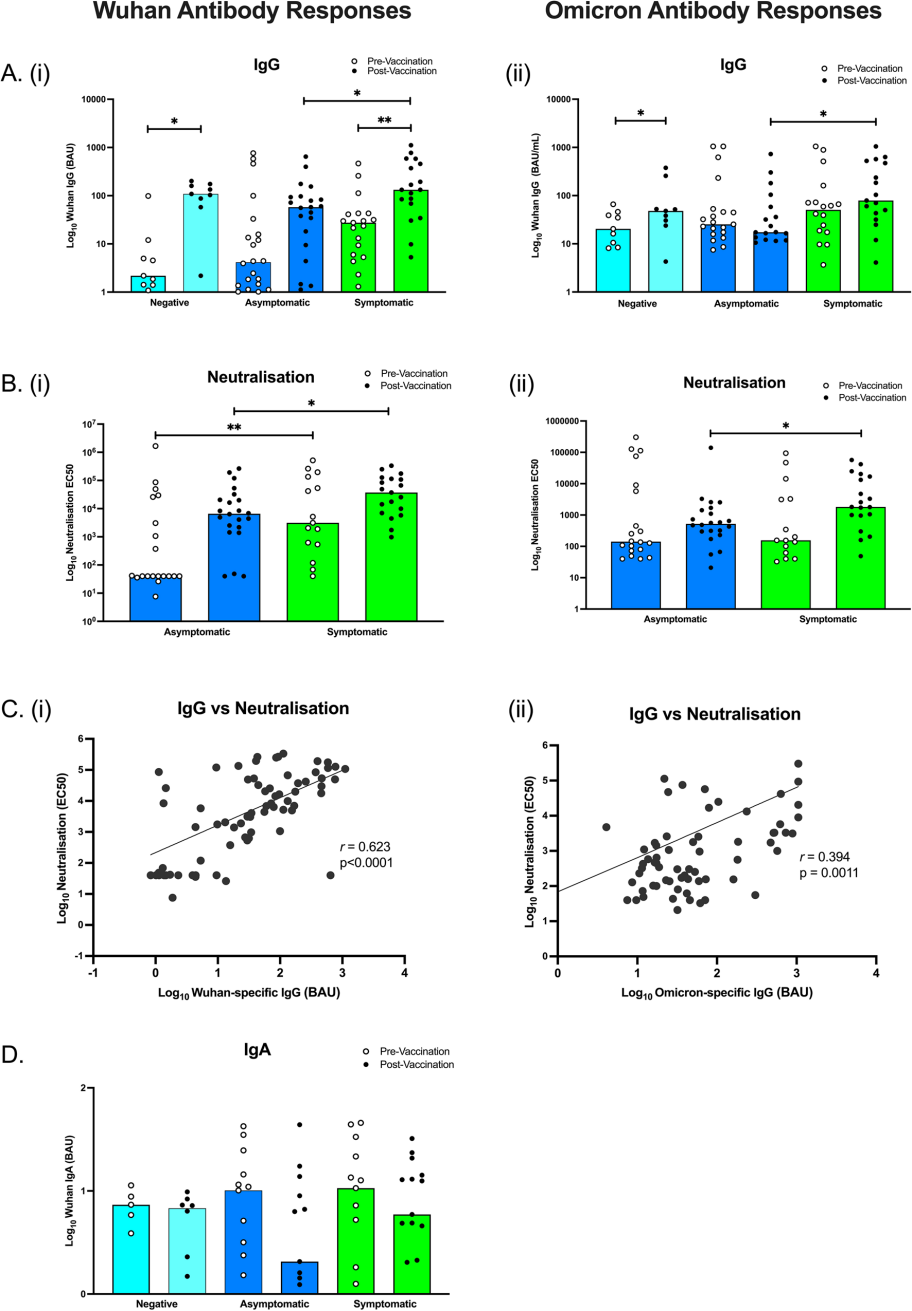
Antibody response to SARS-CoV-2 spike in negative, asymptomatic and symptomatic participants. Wuhan and Omicron-specific IgG and IgA antibody titres were measured by ELISA and a neutralization assay was performed to quantify neutralization titres. Anti-spike IgG antibody responses for Wuhan (Ai) and Omicron (Aii) peptides. Wuhan (Bi) and Omicron (Bii) neutralization antibody responses. The correlation between anti-spike and neutralising antibodies for Wuhan (Ci) and Omicron (Cii). Salivary Wuhan-specific IgA antibody responses (D). Total *N*=102 (9 Negative Unvaccinated, 9 Negative Vaccinated, 23 Asymptomatic Unvaccinated, 23 Asymptomatic Vaccinated, 19 Symptomatic Unvaccinated, 19 Symptomatic Vaccinated). For Wuhan IgA, total *N*=68. Light blue bars = negative participants, dark blue bars = asymptomatic participants, green bars = symptomatic participants. *p<0.05, **p<0.01

Despite trends of higher IgG titres in symptomatic individuals and lowest IgG titres in uninfected participants, there were no significant differences in anti-Wuhan spike IgG antibody responses between the three unvaccinated groups (Fig. 2Ai). Subsequent vaccination resulted in the median anti-Wuhan spike IgG antibody response significantly increasing in negative (*p* = 0.016) and symptomatic participants (*p* = 0.002), but not asymptomatic. Comparisons between the groups identified symptomatic participants had significantly higher Wuhan IgG compared to asymptomatic participants, post-vaccination (*p* = 0.039). Anti-Omicron spike IgG increased post-vaccination in negative participants (*p* = 0.039), but not asymptomatic or symptomatic (Fig. 2Aii). As shown for Wuhan-specific IgG, the median anti-Omicron spike IgG antibody response was also greater in symptomatic participants compared to asymptomatic participants, post-vaccination (*p* = 0.025).

Importantly, the pseudotyped Wuhan antibody neutralization assay showed symptomatic participants had significantly increased neutralization antibodies pre-vaccination (*p* = 0.010) as well as post-vaccination (*p* = 0.026), compared to asymptomatic participants (Fig. 2Bi). In contrast to Wuhan-specific neutralization, there were no differences between pre-vaccinated asymptomatic and symptomatic participants’ Omicron-specific neutralization (*p*>0.05), but there were still differences between post-vaccinated asymptomatic and symptomatic neutralization (*p* = 0.017) (Fig. 2Bii). Furthermore, neutralization correlated with total anti-spike antibody titre (*r* = 0.623, *p* < 0.0001) (Fig. 2Ci). Also as seen in the Wuhan specific responses, Omicron neutralization was correlated to Omicron-specific antibody titre (*r* = 0.394, p = 0.001) (Fig. 2Cii). Wuhan-specific salivary IgA was also analyzed but did not differ between groups pre- or post-vaccination (Fig. 2D). Anti-nucleocapsid antibody titres were also measured and showed no significant differences between pre- and post-vaccination or between groups (Supplementary Figure 3).

### CD4^+^ T cell Responses to Wuhan and Omicron

**Fig. 3:**
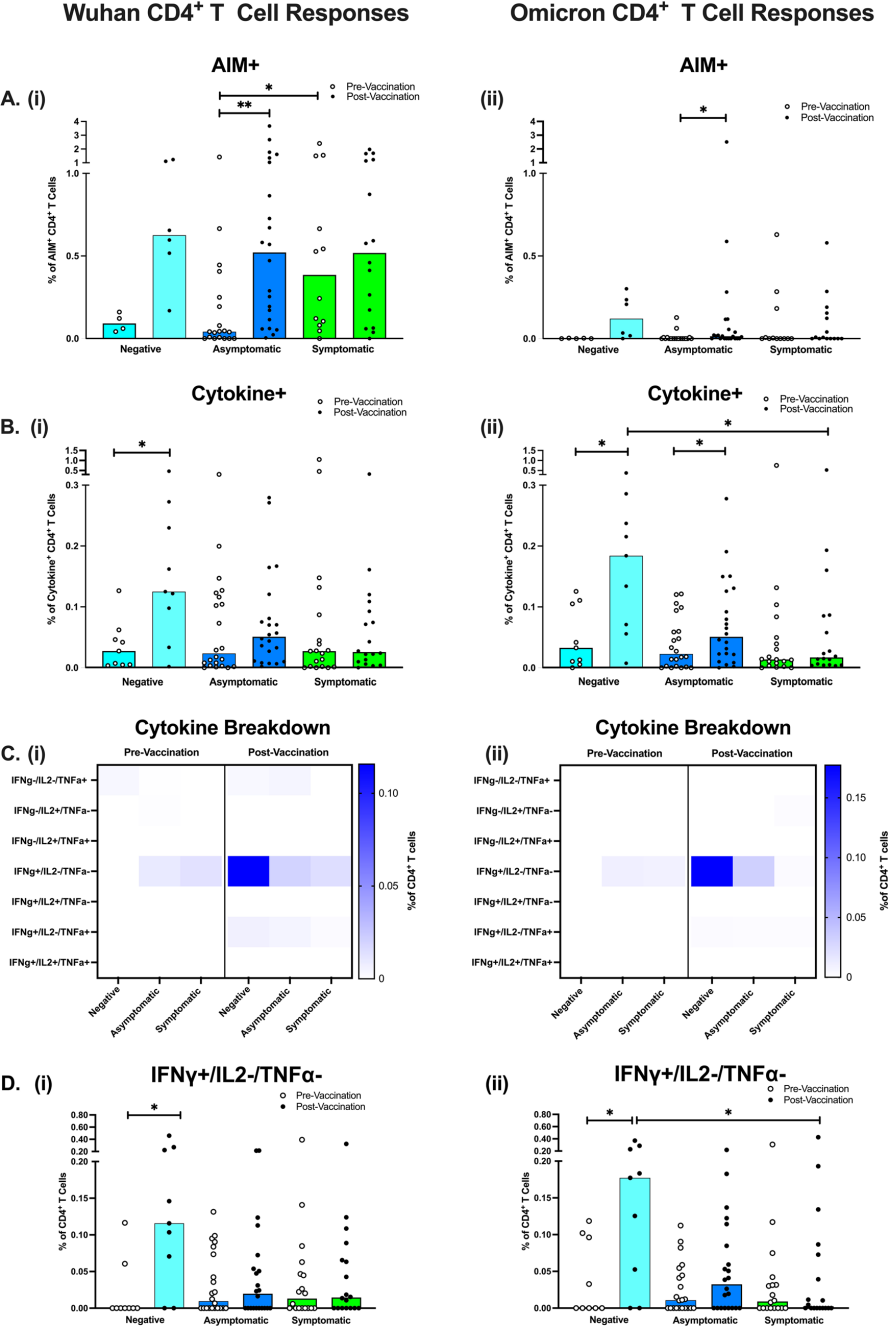
CD4^+^ T cell Responses to SARS-CoV-2 Spike Peptides in Negative, Asymptomatic and Symptomatic Adults. The percentage of activated spike-specific CD4^+^ T cells in response to Wuhan (Ai) and Omicron (Aii) peptides. The total percentage of cytokine-positive CD4^+^ T cells in response to Wuhan (Bi) and Omicron (Bii) spike peptides. Median percent of Wuhan spike-specific cytokine-positive T cells (Ci) and Omicron spike-specific cytokine-positive T cells (Cii). Individual graphs for CD4^+^ T cells producing IFNγ-only are shown to highlight significant differences between groups in response to Wuhan spike (Di) and Omicron spike (Dii). Total *N*=102 (9 Negative Unvaccinated, 9 Negative Vaccinated, 23 Asymptomatic Unvaccinated, 23 Asymptomatic Vaccinated, 19 Symptomatic Unvaccinated, 19 Symptomatic Vaccinated). Light blue bars = negative participants, dark blue bars = asymptomatic participants, green bars = symptomatic participants

CD4^+^ T cell activation markers and cytokine production were measured by flow cytometry after PBMC stimulation with either Wuhan or Omicron-specific peptide pools of the Spike protein. Pre-vaccination, the AIM assay demonstrated there were significantly fewer activated Wuhan spike-specific CD4^+^ T cells in asymptomatic participants compared to symptomatic participants (*p* = 0.029), as well as a trend of fewer activated spike-specific CD4^+^ T cells in negative participants (Fig. 3Ai). Notably, post-vaccination, there were no longer significant differences between symptomatic and asymptomatic activated Wuhan spike-specific CD4^+^ T cells. Vaccination also increased the percentage of activated CD4^+^ T cells across all groups, but only significantly in asymptomatic participants responding to Wuhan spike (*p* = 0.008). In Omicron spike-specific responses, CD4^+^ T cell activation was also low pre-vaccination, but across all groups (Fig. 3Aii). Similar to Wuhan, vaccination increased the percentage of activated Omicron spike-specific CD4^+^ T cells across all groups, but only significantly in asymptomatic participants (*p* = 0.020).

Pre-vaccination, the ICS assay found no significant differences in the percentage of cytokine-producing CD4^+^ T cells between groups, in response to Wuhan or Omicron spike proteins (Fig. 3Bi-Bii). Post-vaccination, the percentage of Wuhan spike-specific CD4^+^ T cells producing cytokines in the negative group significantly increased (*p* = 0.019), but not in participants with previous infection. Similarly, Omicron spike-specific cytokine-positive CD4^+^ T cells significantly increased post-vaccination (*p* = 0.027), but also in the asymptomatic group (*p* = 0.039). Surprisingly, negative participants who had been vaccinated had a significantly higher percentage of cytokine-producing T cells, specific to Omicron spike, compared to symptomatic participants (*p* = 0.015).

The heatmap breakdown of CD4^+^ T cells producing different combinations of cytokines (Fig. 3Ci-Cii) indicates trends of increased IFN-γ-only producing CD4^+^ T cells after vaccination in negative and asymptomatic participants, in response to Wuhan and Omicron spike proteins. Statistical analysis found this was significantly increased post-vaccination in negative participants only, in response to Wuhan (*p* = 0.016) and Omicron (*p* = 0.039) (Fig. 3Dii-Diii). In response to Omicron spike, this IFN-γ only subset was also significantly higher in the negative vaccinated compared to symptomatic vaccinated participants (*p* = 0.047).

Finally, we observed no correlations between IgG responses and CD4^+^ T cell responses (Data can be found in Supplementary Figure 4i and iii).

### CD8^+^ T cell Responses to Wuhan and Omicron

**Fig. 4:**
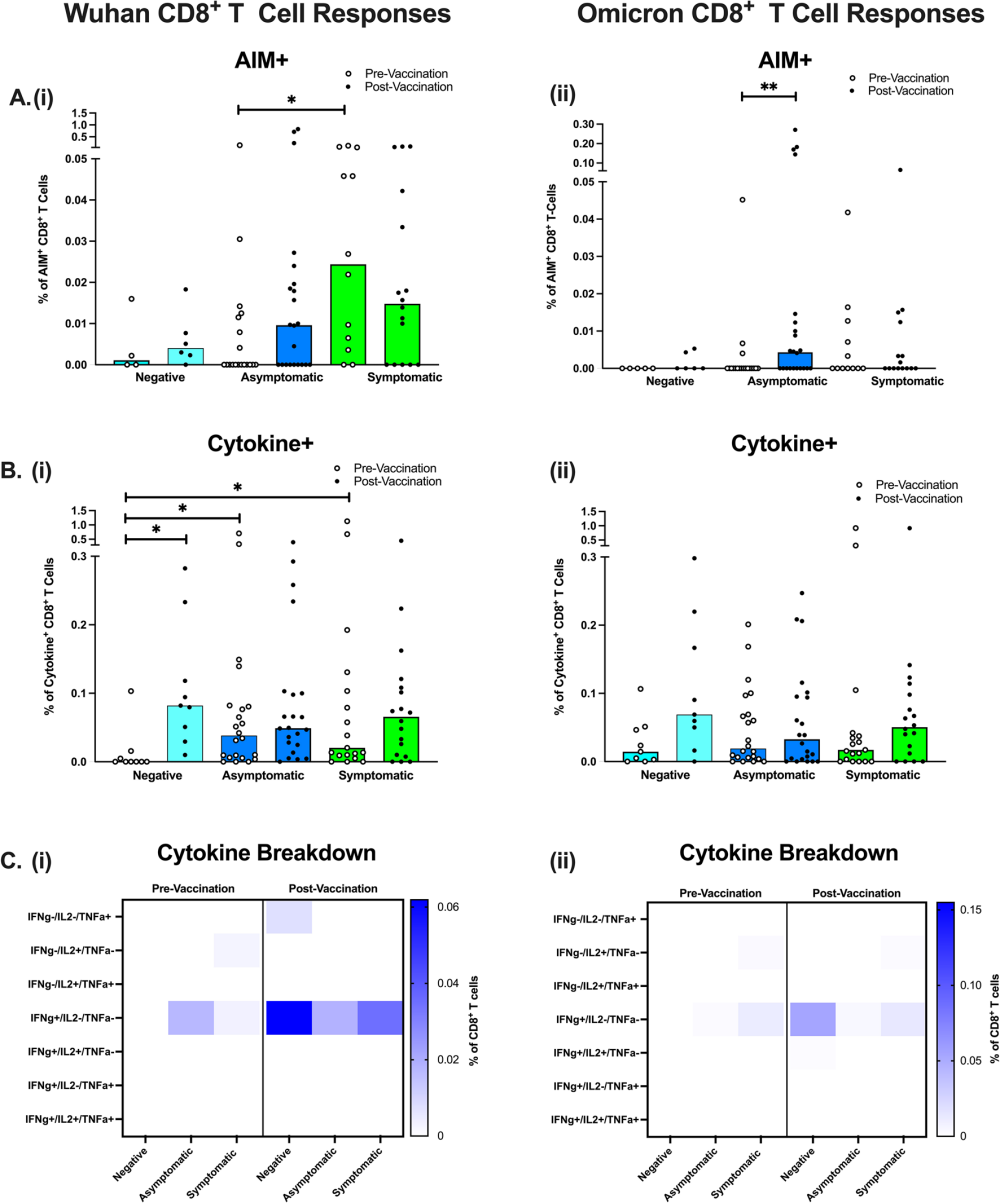
CD8^+^ T cell Responses to SARS-CoV-2 Spike Peptides in Negative, Asymptomatic and Symptomatic Adults. The percentage of activated spike-specific CD8^+^ T cells in response to Wuhan (Ai) and Omicron (Aii) peptides. The percentage of cytokine-positive CD8^+^ T cells in response to Wuhan (Bi) and Omicron (Bii) spike peptides. Cytokine combinations expressed by Wuhan (Ci) and Omicron (Cii) spike-specific CD8^+^ T cells. Total *N*=102 (9 Negative Unvaccinated, 9 Negative Vaccinated, 23 Asymptomatic Unvaccinated, 23 Asymptomatic Vaccinated, 19 Symptomatic Unvaccinated, 19 Symptomatic Vaccinated). Light blue bars = negative participants, dark blue bars = asymptomatic participants, green bars = symptomatic participants

CD8^+^ T cell cytokine production was measured in parallel by flow cytometry after stimulation with the same Wuhan and Omicron spike-specific peptides. As shown in CD4^+^ T cells, the AIM assay demonstrated negative and asymptomatic participants had fewer activated Wuhan spike-specific CD8^+^ T cells compared to symptomatic participants, pre-vaccination. Statistical analyzes reinforced the finding that asymptomatic participants had significantly fewer Wuhan spike-specific CD8^+^ T cells than symptomatic participants (*p* = 0.021). Post-vaccination, there were no significant increases in Wuhan spike-specific activation of CD8^+^ T cells (Fig. 4Ai), but surprisingly, the CD8^+^ T cells did significantly increase after vaccination in response to Omicron spike in asymptomatic participants only (*p* = 0.004) (Fig. 4Aii).

Pre-vaccination, negative participants had significantly fewer Wuhan spike-specific CD8^+^ cytokine-positive T cells, compared to asymptomatic (*p* = 0.035) and symptomatic (*p* = 0.023) participants (Fig 4. Bi). Vaccination significantly increased the percentage of Wuhan spike-specific CD8^+^ T cells producing cytokines in negative participants (*p* = 0.019). In contrast, Omicron spike stimulation resulted in no significant differences pre- or post- vaccination or between groups (Fig 4. Bii).

Analysis of the different cytokines expressed by Wuhan and Omicron spike-specific CD8^+^ T cells (Fig. 4Ci-Cii) showed similar patterns to CD4^+^ T cell cytokine production, where the median percentage of IFN-γ-only producing CD8^+^ T cells was increased in negative vaccinated participants. However, none of these differences between groups reached statistical significance (*p* > 0.05).

Finally, we observed no correlations between IgG responses and CD8^+^ T cell responses (Data can be found in Supplementary Figure 4ii and iv).

### Multi-dimensional Analysis of Wuhan-specific T cells

**Fig. 5:**
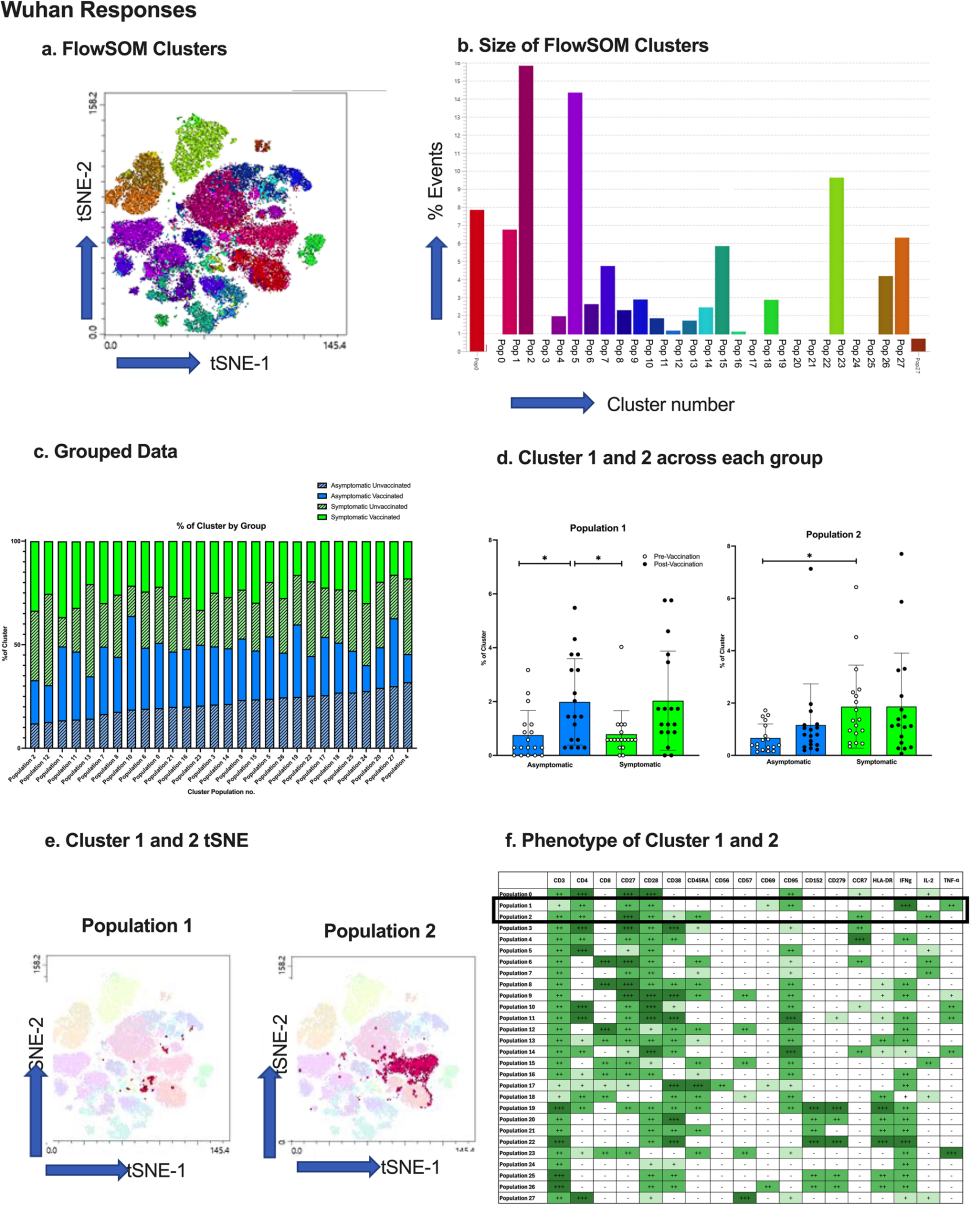
Wuhan-stimulated T cell Clustering Analysis. (**a**) FlowSOM clusters from Wuhan spike-exposed T cells presented as a tSNE plot. (**b**) The number of events in each FlowSOM cluster. (**c**) % of cluster occupied by negative, asymptomatic, and symptomatic subject groups before and after vaccination. (**d**) Statistical analysis of the differences between clusters 1 and 2, between groups. (**e**) tSNE plots of cluster 1 and 2 to identify location and size of cluster. (**f**) T cell marker expression of clusters 1 and 2. *=*p*<0.05, **=*p*<0.01, *N*=19**.** Light blue bars = negative participants, dark blue bars = asymptomatic participants, green bars = symptomatic participants

Multi-dimensional analysis was performed as an unbiased analysis of Wuhan-specific, cytokine-producing T cells, to investigate whether there were any differences which were not identified by conventional flow analysis. The multidimensional clustering analysis, using FlowSOM, grouped the Wuhan-stimulated PBMCs into a total of 28 clusters with different expression markers (Fig. 5a-b). To determine if there were any differences between cytokine-positive T cell phenotypes of subjects following Wuhan spike peptide exposure, the percentage of cells present from each subject group in each cluster was measured (Fig. 5c). Statistical analysis found two clusters (Fig. 5d) were significantly different between groups, and their tSNE plots are presented (Fig. 5e).

A two-way ANOVA indicated the cell population ‘Cluster 1’ was significantly higher in asymptomatic vaccinated than asymptomatic unvaccinated and symptomatic unvaccinated adults (*p*<0.05). Despite trends of symptomatic vaccinated increased compared to unvaccinated, this was not statistically significant. To identify the phenotype of the cluster, Cluster Explorer analysis was run (Fig. 5f), and the results indicate cluster 1 was CD4^+^ effector memory (CCR7^-^/CD45RA^+^) cells which expressed CD27, CD28, CD95, CD69, and produced IFN-γ and TNFα (but not IL-2).

In addition, the cell population ‘Cluster 2’ was significantly higher in symptomatic participants compared to asymptomatic participants, pre-vaccination (*p*<0.01), but post-vaccination there were no significant differences. Cluster 2 was identified as CD4^+^ T cells with a naïve phenotype (CD454RA^+^/CCR7^+^/CD27^+^/CD28^+^), expressing CD152, CD38, and producing IL-2 only.

### Multi-dimensional Clustering Analysis of Omicron-specific T cells

**Fig. 6:**
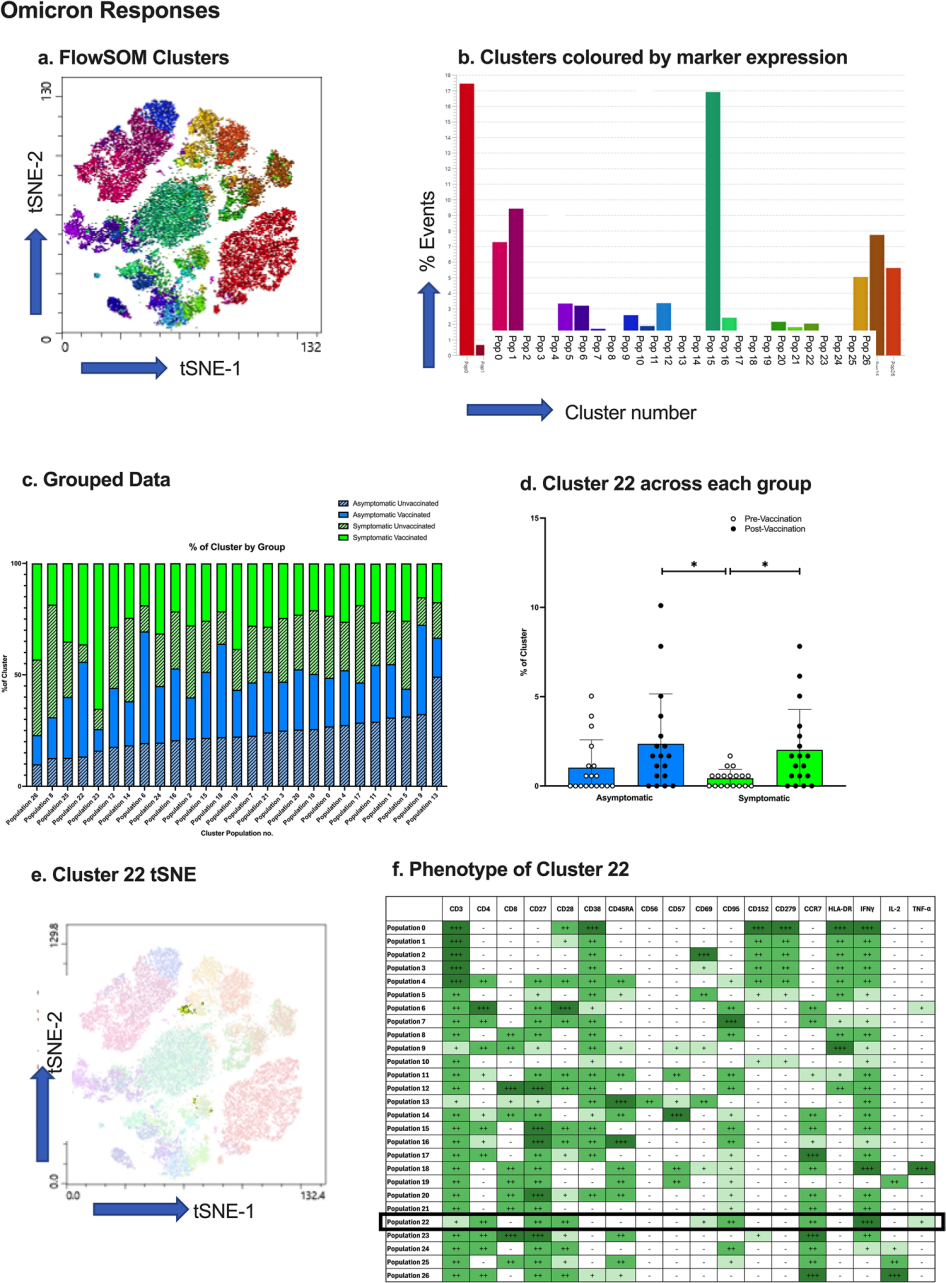
Omicron-stimulated T cell Clustering Analysis. (**a**) FlowSOM clusters from Omicron spike-exposed T cells presented as a tSNE plot. (**b**) The number of events in each FlowSOM cluster. (**c**) % of cluster occupied by negative, asymptomatic, and symptomatic subject groups before and after vaccination. (**d**) Statistical analysis of the differences between cluster 22 between groups. (**e**) tSNE plots of cluster 22 to identify location and size of cluster. (**f**) T cell marker expression of cluster 22. *=*p*<0.05, *N*=19

Clustering analysis of Omicron spike-specific, cytokine-producing T cells identified 27 meta-clusters with different expression markers (Fig. 6a-b). The percentage of cells present from each participant group in each cluster was measured (Fig. 6c), and a two-way ANOVA found one cluster (Fig. 6d) was significantly different between groups, of which the cluster tSNE plot is presented (Fig. 6e). The results indicated the cells in Cluster 22 were significantly higher in asymptomatic (*p* = 0.0448) and symptomatic (*p* = 0.042) participants post-vaccination, compared to pre-vaccination. Cluster Explorer analysis helped identify the phenotype of cluster 22 (Fig. 6f), which was CD4^+^ effector memory T cells (CD45RA^-^/CCR7^+^) producing high amounts of IFNγ and low TNFα, as well as the expression of CD28, CD27 and CD95, notably similar to Cluster 1 from Wuhan clustering analysis.

A combined multi-dimensional analysis of both Wuhan and Omicron T cells was performed to decipher whether peptide-responsive cells form the same clusters. The clustering analysis (found in Supplementary Figure 5) shows 33 clusters, and a two-way ANOVA found no significant differences between Wuhan and Omicron for each cluster (*p*>0.05).

## Discussion

To investigate long-term immunity to SARS-CoV-2, measuring T cell responses in addition to antibody responses is crucial, as T cells underpin long-lasting immunity. Despite existing studies investigating the immune response in asymptomatic disease [14, 15, 20–22], asymptomatic infection is less well-characterised, especially in a largely young and healthy demographic, and often in the absence of a true asymptomatic diagnosis. Thus, this well-defined, cohort study recruited young asymptomatic adults, identified by an established asymptomatic testing service across 3 UK centres, and age/sex-matched symptomatic and uninfected controls. We show deep-phenotyping of T cell responses, in addition to analysing antibody titres, together demonstrate asymptomatic young adults exhibit decreased humoral and cellular responses to natural SARS-CoV-2 infection, compared to those with symptomatic infection.

Traditionally, systemically administered vaccines are thought to generate strong IgG responses, which provide strong protection against lower respiratory tract disease [23, 24]. In agreement with this previous research, levels of IgG specific to Wuhan spike proteins increased after vaccination in all subject groups, but interestingly, not significantly in asymptomatic participants. This lack of significance may be due to the large variation in antibodies detected prior to vaccination in this group. Weaker IgG responses in vaccinated asymptomatic individuals were further reinforced by significantly lower Wuhan and Omicron-specific IgG titres, compared to those of vaccinated symptomatic participants. Despite participant samples being obtained before the Omicron variant emerged, Omicron-specific IgG also significantly increased after vaccination, but only in the uninfected (negative) group, indicating some ability of the vaccine to protect against future SARS-CoV-2 strains. Existing literature found primary vaccination with current COVID-19 vaccines and previous SARS-CoV-2 infections offered low protection against Omicron BA.1 and BA.2 infection [25]. Along with IgG playing an important role in humoral immunity, IgA has also been shown to have an important early role in the neutralization of SARS-CoV-2 virus after infection [26]; here, we show the presence of IgA after infection in all groups, but vaccination did not increase IgA titres. In fact, although not statistically significant, IgA titres slightly decreased after vaccination. This is consistent with previous literature which found salivary IgA does not increase after 2 doses of the mRNA SARS-CoV-2 vaccine [27]. Despite lowest SARS-CoV-2 specific antibody levels in uninfected individuals, the lack of significant differences between uninfected and asymptomatic or symptomatic participants are likely due to the presence of existing antibodies from previous seasonal coronavirus exposure, indicating a cross-reactivity with SARS-CoV-2 peptides [28–30].

In addition to weaker IgG responses, asymptomatic participants also exhibited fewer Wuhan-specific neutralization antibodies pre- and post-vaccination, as well as fewer Omicron-specific neutralization antibodies post-vaccination, which is also demonstrated in previous research [15, 31–33]. There is a protective effect of neutralization antibodies against future re-infection [34], suggesting symptomatic individuals could be better protected against SARS-CoV-2 re-infection compared to asymptomatic individuals. As expected, both Wuhan and Omicron neutralising antibodies positively correlated to total anti-spike antibody titre. Overall, the antibody response results are congruent with existing literature [35–37], which states symptomatic individuals produce a greater magnitude of antibody response, compared to asymptomatic individuals. Thus, despite this being a young and healthy cohort, asymptomatic infection still resulted in a weaker humoral response to SARS-CoV-2, before and after vaccination, implying symptomatic cases could be more likely to provide increased protective immune responses following infection and vaccination. In addition, asymptomatic individuals have reduced humoral protection against future strains, as the responses to Omicron were lower than symptomatic IgG and neutralization responses.

Compared to antibodies, T cells mediate a faster and more potent response to SARS-CoV-2 [11], thus, cognate T cell immune responses before/after vaccination were also analyzed. Asymptomatic individuals have been shown to produce an efficient memory T cell response to SARS-CoV-2 during the convalescent phase [14, 20]. However, conventional flow cytometric analysis of Wuhan spike-stimulated T cells indicated unvaccinated asymptomatic participants had significantly fewer responding CD4^+^ and CD8^+^ T cells, similar to that of the uninfected young adults. This highlights asymptomatic infection results in a reduced cellular response to SARS-CoV-2, which could be explained by a more effective immune response during initial exposure, resulting in this less robust response after exposure. Although these differences dissipated after vaccination, resulting in no significant differences between groups post-vaccination. Thus, vaccination is key in providing a more robust cellular response in asymptomatic individuals. In agreement with Sekine et al. [20], T cell cytokine analysis indicated CD4^+^ and CD8^+^ T cells producing IFN-γ only was the dominant response in both asymptomatic and symptomatic adults. Surprisingly, the percentage of activated and cytokine-producing CD4^+^ T cells was highest in negative vaccinated individuals, in response to Wuhan and Omicron. These observations were likely due to CD4^+^ T cells producing IFN-γ only, which were significantly increased post-vaccination in negative participants in response to Wuhan and Omicron. This indicates vaccination gives a differential immune response compared to natural infection.

As previously mentioned, the participants were infected prior to the Omicron outbreak. Thus, it is unsurprising that Omicron-specific activation of CD4^+^ and CD8^+^ T cells were reduced compared to Wuhan-specific stimulation. These reduced responses predict a lack of cellular immunity to future variants of SARS-CoV-2, despite previous infection or vaccination. These results are supported by a study of Wuhan-infected health-care workers who were subsequently triple-vaccinated, showing a lower magnitude of T cell responses to the Omicron spike peptide than individuals who had not been initially infected with Wuhan [38]. These results suggest immunological imprinting through exposure to the ancestral strain of SARS-CoV-2 impairs the cross-reactivity of the response to emerging variants of SARS-CoV-2. In addition, Omicron variants can bind in a separate register to the HLA-II heterodimer and abrogate T cell responses, which could further explain these results [39]. In contrast, we show a combined multi-clustering analysis of T cells responding to both Wuhan and Omicron found no significant differences in phenotype between Wuhan and Omicron-responsive T cells. In addition, prior research indicates no differences in T cell responses to Wuhan or Omicron, suggesting T cells generated in response to vaccination or previous SARS-CoV-2 infection can cross-recognize Omicron [40].

Multi-dimensional clustering analysis pinpointed additional populations that differed between groups, highlighting the importance of unbiased multi-dimensional analysis. Omicron-specific CD4^+^ effector memory T cells producing high amount of IFN-γ and low TNFα were significantly higher in symptomatic vaccinated individuals compared to symptomatic unvaccinated individuals. A similar cluster was identified after exposure to Wuhan, but higher in asymptomatic vaccinated compared to unvaccinated individuals. This is as expected as this suggests this group of CD4^+^ effector memory T cells increases after vaccination, which is representative of a long-lived immune response, characteristic after vaccination [11]. Notably, a population of CD4^+^/CD45RA^+^/CCR7^+^ T cells were also identified by clustering analysis as significantly different among groups, where symptomatic unvaccinated adults had a significantly higher percentage of activated CD4^+^/CD45RA^+^/CCR7^+^ T cells producing IL-2, compared to asymptomatic unvaccinated adults. This population may be naïve CD4^+^ T cells, which are crucial in SARS-CoV-2 peptide recognition by antigen presenting cells, and produce IL-2 to differentiate into memory cells [41]. Thus, an increased population in symptomatic adults indicates a higher potential to generate CD4^+^ memory cells, and subsequently a longer-lasting immune memory. Furthermore, research suggests this population may in fact be revertants; CD4^+^ memory T cells reverting back to a ‘naïve’ phenotype upon activation [42–44], as unlike conventional naïve T cells, this CD4^+^ subset are activated and producing IL-2.

In conclusion, utilising this well-defined young adult cohort, we identify key differences in both SARS-CoV-2 humoral and cellular immunity, dependent on the presence of symptoms during infection. We demonstrate asymptomatic young adults present decreased antibody and T cell responses to Wuhan and Omicron, pre-vaccination. Post-vaccination, antibody responses are still inferior, but T cell immunity resolves to levels seen in the symptomatic group, highlighting the requirement of targeted vaccines to improve antibody and T cell responses following asymptomatic SARS-CoV-2 infection.

### Supplementary Information

ESM 1 (DOCX 3170 kb)

## Data Availability

All data required to evaluate the conclusions in the paper are present in the manuscript or its appendix. Further information on the study protocol or de-identified datasets generated and analyzed within this publication are available from the corresponding author on reasonable request.
