# Supplementary material for: Lower Humoral and Cellular Immunity Following Asymptomatic SARS-CoV-2 Infection Compared to Symptomatic Infection in Education (The ACE Cohort)
Source: J Clin Immunol. 2024 Jun 10;44(6):147. doi: 10.1007/s10875-024-01739-0 (PMC11164737; doi:10.1007/s10875-024-01739-0)
Supplement: Supplementary file 1 — (DOCX 3170 kb) [file 10875_2024_1739_MOESM1_ESM.docx]

**Supplementary Material**

**Supplementary Methods**

***Antibody titre assays***

Heparinized whole blood was centrifuged at 300 xg for 8 minutes and the upper plasma containing layer removed and further centrifuged at 800 xg for 5 minutes. Plasma was tested using two separate in-house ELISAs for detection of Wuhan and Omicron-specific spike antibodies. In each case, 384 well Maxisorp (NUNC) assay plates were coated with 1µg/mL of either Wuhan strain SARS-CoV-2 full length spike protein (His tagged, CHO expressed, Native Antigen Company, UK) or Omicron strain SARS-CoV-2 full length spike protein (His tagged, Baculovirus expressed, Sino Biological, Stratech UK), in carbonate-bicarbonate buffer (CBC; Merck). Plates were sealed with foil film and incubated overnight at 4°C before washing 3 times with PBS + 0.05% Tween 20 (PBS-T) using a Biochrom ASYS Atlantis plate washing robot. Wells were then immediately filled with blocking solution (3% whey powder (w/v) in PBS containing 0.05% Tween 20, 0.05% sodium azide, and 0.01% EDTA) and blocked overnight at 4°C. Plates were washed a further 3 times before 1:120 diluted plasma samples (in 3% whey blocking solution) was added in duplicate wells. SARS-CoV-2 antibody positive and negative serum controls were obtained from the National Institute of Biological Standards and Controls (NIBSC, UK), using a WHO reference panel (NIBSC code: 20/268). Each assay also contained a 12-point standard dilution of NIBSC 20/162 positive standard diluted two-fold from 1:120, two negative controls from the NIBSC assay verification panel, and the QC standard (20/764) all also diluted at 1:120. After incubating for one hour at room temperature, the plate was washed 3 times, and gamma chain-specific anti-human IgG HRP conjugate (Sigma, A0170) was added at a 1:30,000 dilution. This was incubated for 30 minutes at room temperature. Following a final three washes, One-step Ultra-3,3′,5,5′-tetramethylbenzidine (TMB) substrate solution (ThermoFisher Scientific) was added to each well for 20 minutes at room temperature. Finally, 2N H_2_SO_4_ was added to each well and absorbance read at 450 and 600nm using an EPOCH microplate reader (BioTek, UK). Data were presented as a conversion of delta OD (450nm-600nm) into BAU (binding antibody units). All assays were performed on Opentrons OT-2 liquid handling robots.

***Virus neutralization assays***

Pseudotyped viruses were neutralised by incubating with serially diluted, heat-inactivated human plasma samples for 1 h at 37°C. Cell only and virus + cell only controls were included. After 1 h, HeLa-ACE2 cells were added to each well. Following 48 h of incubation at 5% CO_2_ and 37°C, luminescence was measured using the BrightGlo Luciferase Assay System (Promega, UK). Neutralization was calculated relative to the virus + cell and cell only controls. Data was analyzed in GraphPad Prism where 50% neutralization (ID50) values were calculated and the limit of detection for neutralization was set at an ID50 of 20. Within each group, the ID50 values were summarized as a geometric mean titer (GMT).

***Statistical analysis***

Data analysis for antibody responses was performed using Graphpad Prism (V9.3.0). Data analysis for flow cytometry was performed using Kaluza version 2.2 (Beckman Coulter, Indianapolis, IN, USA). Exported statistics were then analyzed using Graphpad Prism (V9.3.0). T cell data was calculated as background subtracted data. Background subtracted data was calculated as the frequency of cells in the antigen stimulation minus the frequency in the unstimulated condition.

All data were not normally-distributed, as defined by Kolmogorov-Smirnov test. A simple linear regression analysis was performed for comparing neutralization and antibody titres. Wilcoxon matched-pairs signed rank tests were performed to compare pre-vaccination and post-vaccination differences within each group. A Kruskal-Wallis test with Dunn’s Multiple Comparisons test was performed to compare differences between groups.

Data generated from the multi-dimensional clustering analysis was normally-distributed, so a two-way ANOVA was conducted to compare the percentage of group-specific cells present in each cluster.

**Supplementary Figure 1**

**
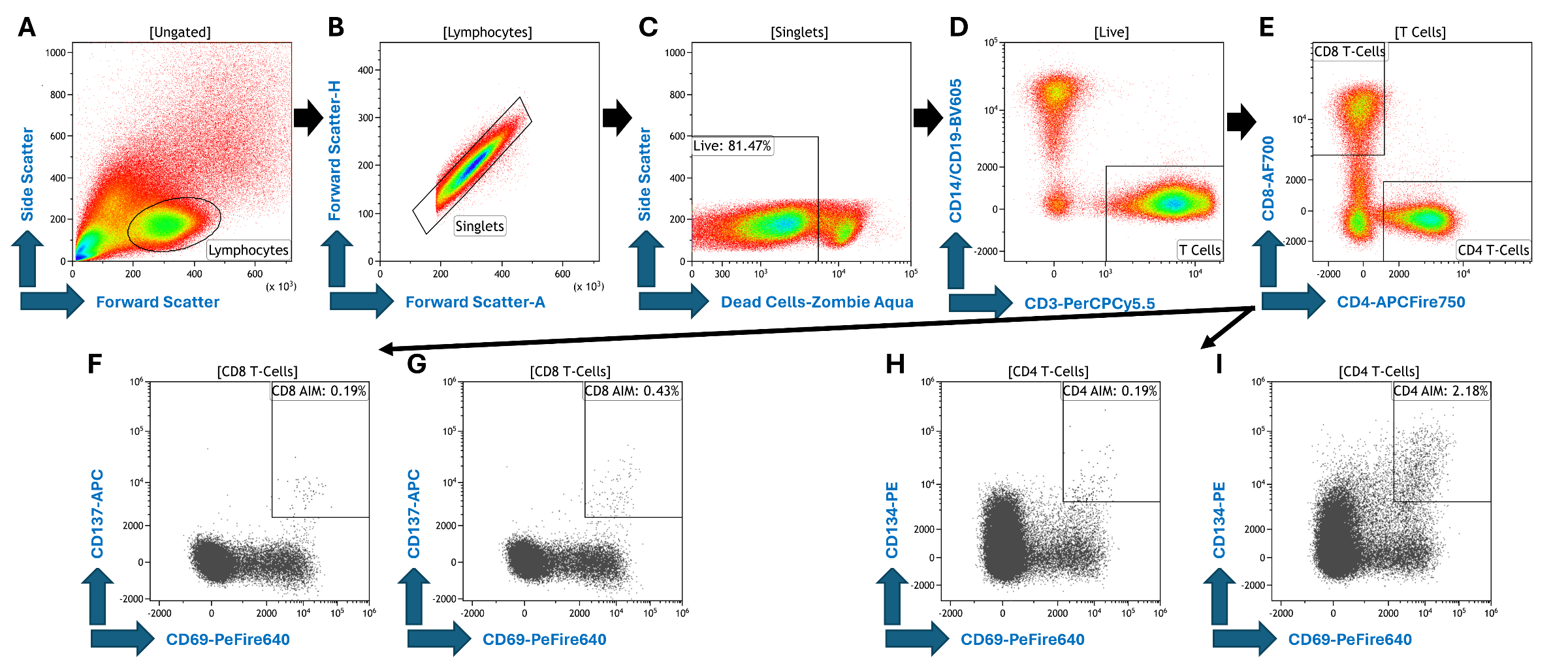
**

**Supplementary Figure 1. Flow Cytometric Gating Strategy for AIM Assay.** (A) Lymphocytes were gated by forward and side scatter profile and (B) single cells were identified by height vs area analysis. (C) Viable zombie aqua low cells were gated and (D) CD3^+^/CD14^-^/CD19^-^ T cells identified for further dissection into (E) CD4 and CD8 T cells. Activation induced markers for CD8 T cells are defined as CD69^+^/CD137^+^ in (F) unstimulated cells and (G) Wuhan spike peptide pool stimulated cells. Activation induced markers for CD4 T cells are defined as CD69^+^/CD137^+^ in (H) unstimulated cells and (I) Wuhan spike peptide pool stimulated cells. Example shown is donor FF07732593.

**Supplementary Figure 2**

**
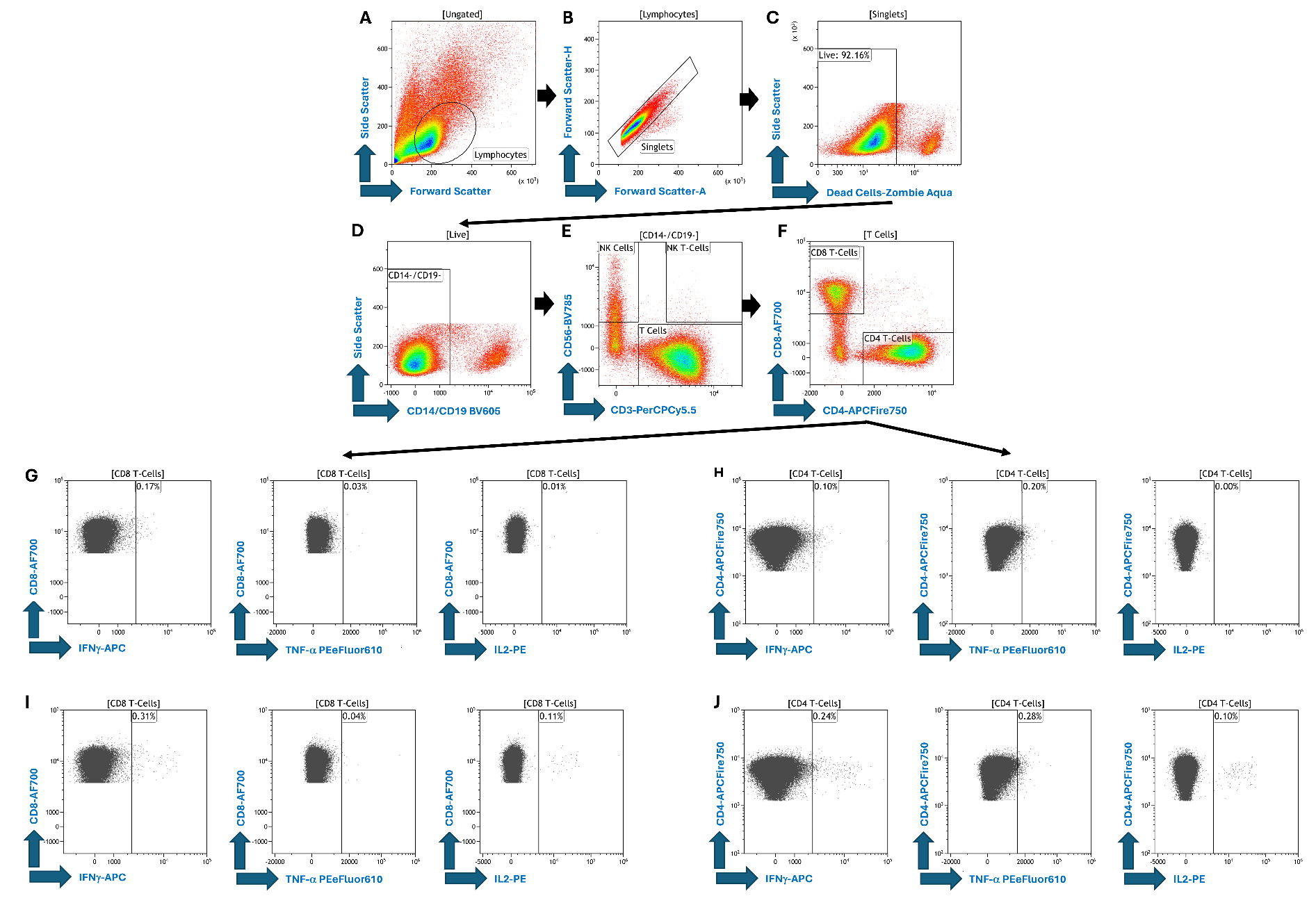
**

**Supplementary Figure 2. Flow Cytometric Gating Strategy for ICS Assay.** (A) Lymphocytes were gated by forward and side scatter profile and (B) single cells were identified by height vs area analysis. (C) Viable zombie aqua low cells were gated and (D) CD14^-^/CD19^-^ were gated to remove monocytes and B Cells and (E) CD3^+^/CD56^-^ T-cells identified for further dissection into (F) CD4 and CD8 T cells. IFNγ, TNF-α and IL-2 positive cells were enumerated in: (G) unstimulated CD8 T cells, (H) unstimulated CD4 T cells, (I) Wuhan spike peptide pool stimulated CD8 T cells, and (J) Wuhan spike peptide pool stimulated CD4 T cells. Example shown is donor CNN025-1.

**Supplementary Table 1**

**Supplementary Table 1.** P-values for demographic comparisons between groups.

|  | **Asymptomatic vs. Symptomatic** | **Asymptomatic vs. Negative** | **Symptomatic vs. Negative** | **Statistical Test** |
| --- | --- | --- | --- | --- |
| **Age** | P > 0.9999 | P = 0.9317 | P > 0.9999 | Kruskal Wallis with Dunn’s multiple comparisons |
| **Ethnicity** | P = 0.9378 | P = 0.2067 | P = 0.4392 | RM one-way ANOVA with Tukey multiple comparisons |
| **Days since vaccination** | P > 0.9999 | P > 0.9999 | P = 0.6852 | Kruskal Wallis with Dunn’s multiple comparisons |
| **Type of Vaccine** | P = 0.9101 | P = 0.4516 | P = 0.1231 | RM one-way ANOVA with Tukey multiple comparisons |
| **Days between visits** | P = 0.8562 | P = 0.0554 | P = 0.4237 | Kruskal Wallis with Dunn’s multiple comparisons |

**Supplementary Figure 3**

**
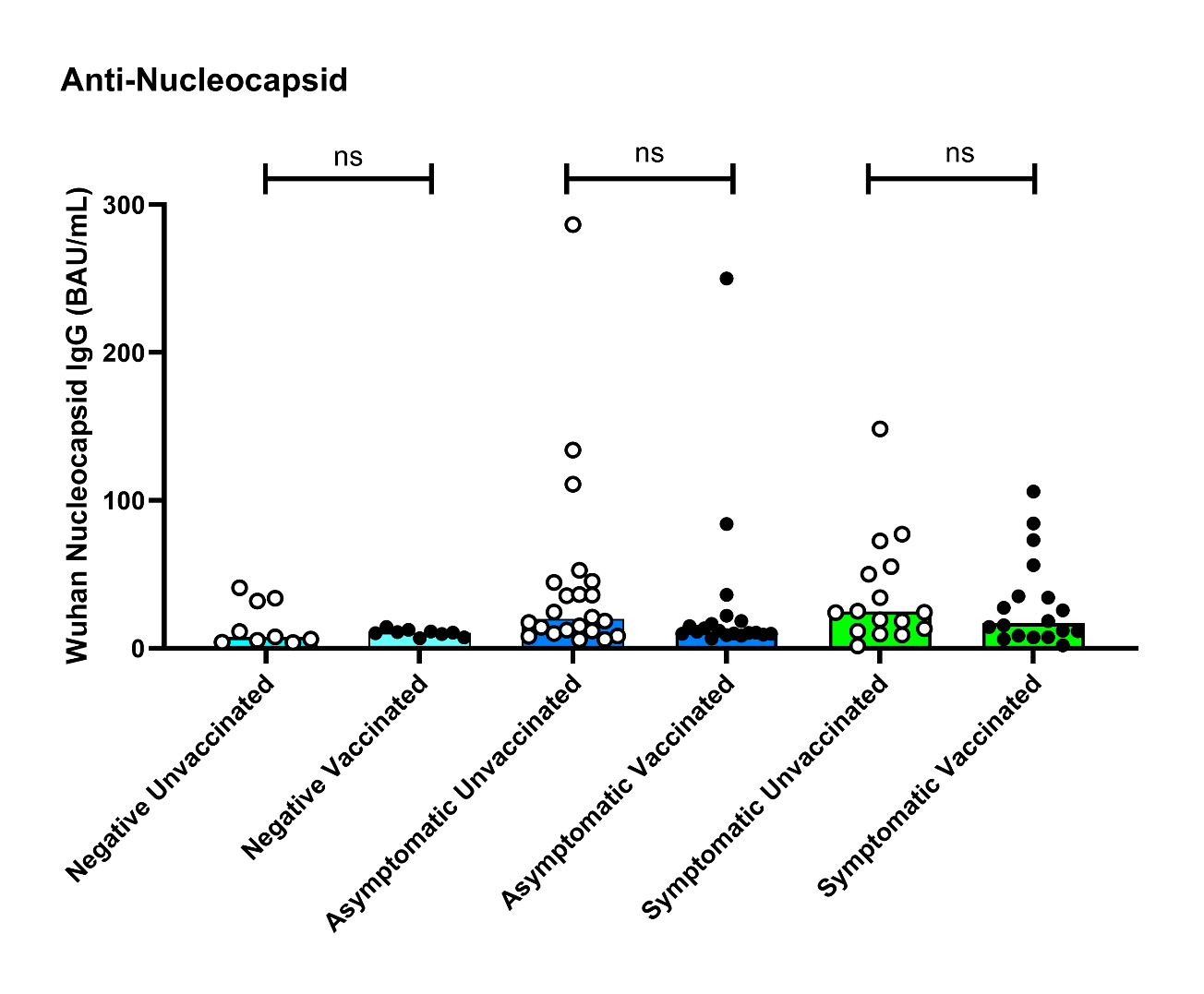
**

**Supplementary Figure 3. Antibody response to SARS-CoV-2 nucleocapsid in negative, asymptomatic and symptomatic participants.** Wuhan nucleocapsid-specific IgG antibody titres were measured by in-house ELISA. Wilcoxon matched-pairs signed rank tests were performed to compare pre-vaccination and post-vaccination differences within each group. ns (p > 0.05). Negative (n=9), asymptomatic (n=23), symptomatic (n=19).

**Supplementary Figure 4**


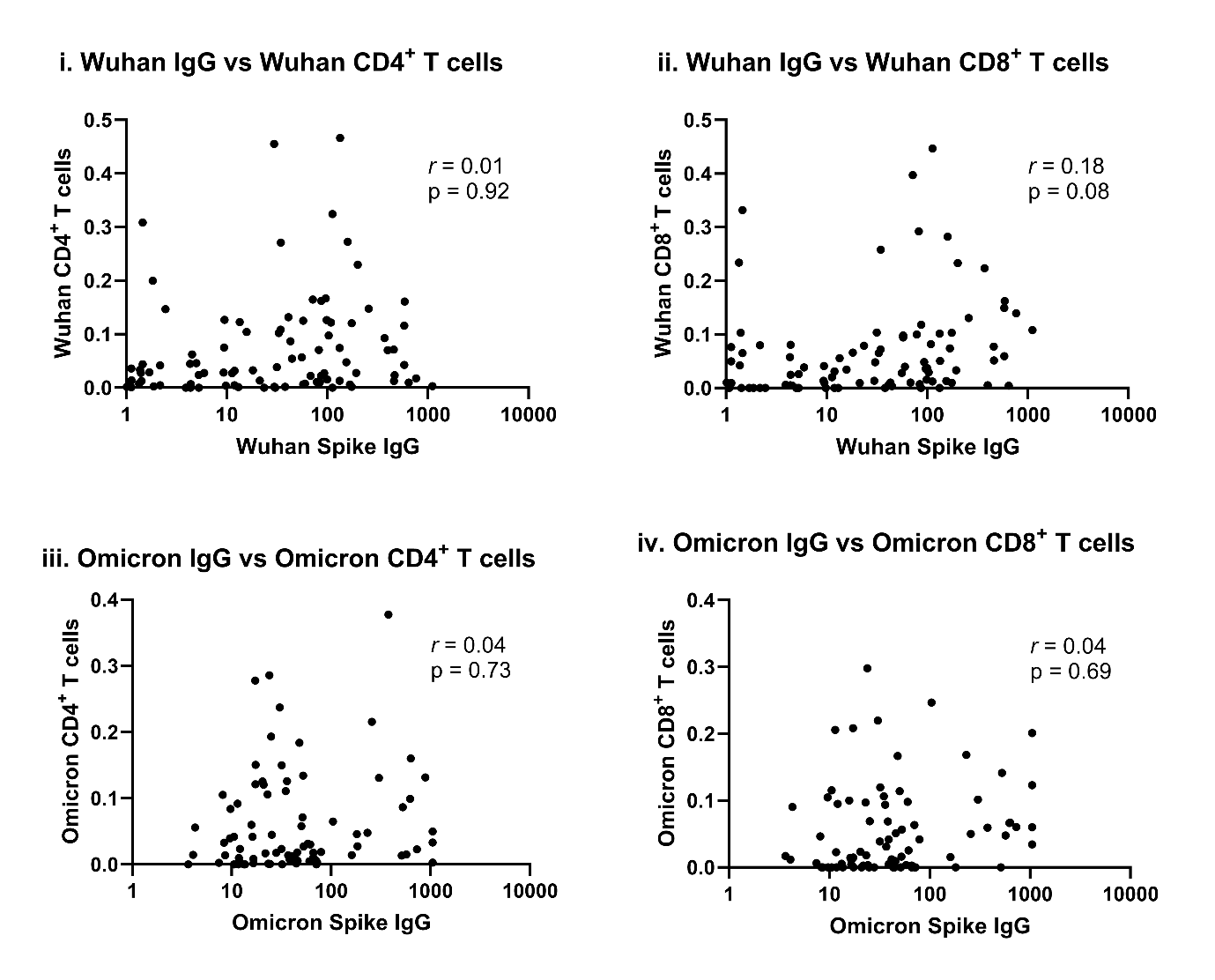


**Supplementary Figure 4. Humoral and cellular correlations for Wuhan and Omicron spike.** Pearson’s r correlations were performed to compare between (i) Wuhan spike IgG and CD4^+^ T cells producing cytokines, (ii) Wuhan spike IgG and CD8^+^ T cells producing cytokines, (iii) Omicron spike IgG and CD4^+^ T cells producing cytokines, and (iv) Omicron spike IgG and CD8^+^ T cells producing cytokines. N= 102.

**Supplementary Figure 5**

**
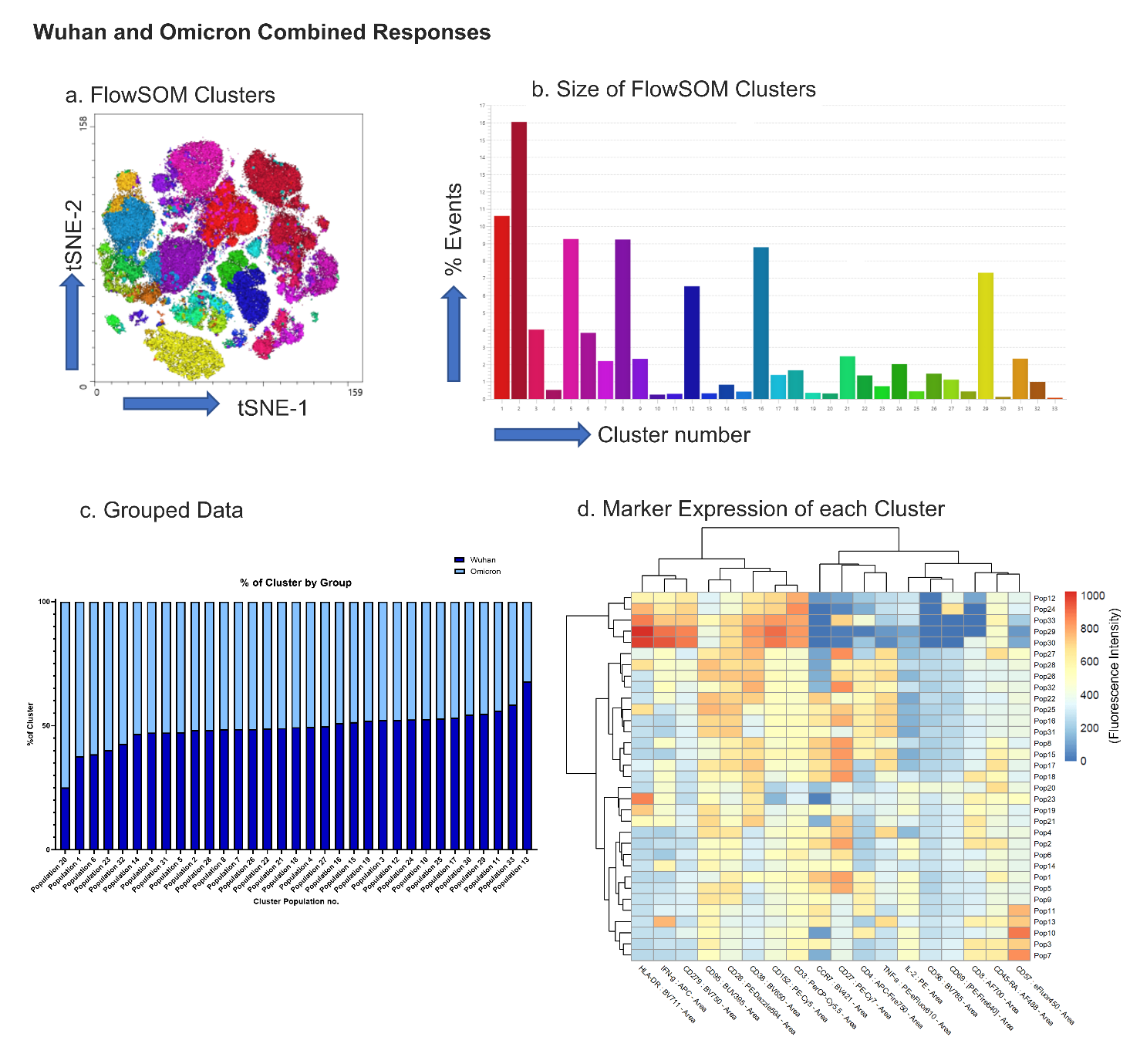
**

**Supplementary Figure 5.** Combined Wuhan and Omicron clustering analysis. (a) FlowSOM clusters from Omicron and Wuhan spike-exposed T cells presented as a tSNE plot. (b) The number of events in each FlowSOM cluster. (c) % of cluster occupied by Wuhan T cells and Omicron T cells. (d) T cell marker expression of each cluster. N=3.
